# The mechanism of intestinal flora dysregulation mediated by intestinal bacterial biofilm to induce constipation

**DOI:** 10.1080/21655979.2021.1973356

**Published:** 2021-09-13

**Authors:** Ruibiao Fu, Zhongpeng Li, Rui Zhou, Chaoyang Li, Shuai Shao, Jin Li

**Affiliations:** Department of Gastrointestinal Surgery, Xuzhou Municipal Hospital Affiliated to Xuzhou Medical University, Xuzhou, Jiangsu Province, China

**Keywords:** Intestinal bacterial biofilm, dysregulation of intestinal flora, constipation, protein concentration detection

## Abstract

To explore mechanism of intestinal flora dysregulation promoting constipation, 60 specific pathogen-free (SPF) mice were used as research objects and were treated with constipation population fecal fluid gavage and distilled water gavage. Then, relationship between intestinal dysregulation and constipation in mice with biofilm-mediated intestinal flora was investigated in vitro. The results showed that recombinant serotonin transporter (SERT) messenger ribonucleic acid (mRNA) level of the constipation population fecal fluid gavage group and the relative expression level of SERT mRNA were 1.61 ± 0.08 and 1.49 ± 0.06, which were higher markedly than those of distilled water group (*P* < 0.05). The level of 5-hydroxytryptamine (5-HT) in colonic tissue of the constipation population fecal fluid gavage group was 145.36 ± 14.12 ng/mL, and the expression level of 5-HT on the surface of epithelial cells of biofilm-positive colonic tissue was 20.11 ± 2.03, which were significantly lower than those of the distilled water group, with statistical significance (*P* < 0.05). Besides, the microbial sequencing of fecal flora indicated that The Akk and bacteroidetes ofconstipation population fecal fluid gavage group were higher hugely than those of distilled water group (*P* < 0.05).In conclusion, after the occurrence of constipation, the diversity of intestinal microflora decreased, and the probiotics reduced. Iintestinal microflora dysregulation would lead to increase of SERT expression level in defecation function and intestinal motility in mice, and the decrease of 5-HT, thereby changing the intestinal movement resulting in mucosal protective barrier damage,thereby causing changes in intestinal movement and the destruction of the intestinal mucosal protective barrier, which eventually resulted in constipation. The occurrence of constipation could be improved by regulating balance of intestinal flora, increasing the diversity of flora, and reducing the genus of opportunistic pathogens.

## 1. Introduction

Constipation is a typical clinically refractory disease, which can be divided into organic constipation and functional constipation according to its causes. Organic constipation is usually caused by organic diseases of the organs, such as colon tumor, pelvic or abdominal tumor, rectum or anus disease, and diabetes mellitus [1,2]. Functional constipation refers to a functional disease caused by non-organic lesions with typical characteristics of long duration, difficult defecation, less frequency, and dry and hard stool [3]. Different from organic constipation, the causes of functional constipation are mostly related to patients’ eating habits and living environment, and so on. In addition, drug abuse can also cause functional constipation [4]. The incidence of functional constipation is high, and it is difficult to treat. Long-term constipation will not only seriously affect patients’ quality of life but may even induce gastrointestinal inflammation, hypertension, and other cardiovascular diseases, causing great troubles to the life of patients [5].

Studies have found that most patients with constipation are usually accompanied by the disorder of intestinal flora. When the dynamic balance of the intestinal flora is destroyed, the metabolism of the body is affected, and a series of pathophysiological changes occur in the body, which aggravates constipation [6]. The normal intestinal contains more than 400 bacteria, including physiological bacteria, pathogenic bacteria, and a variety of pathogenic bacteria. Under normal circumstances, these bacteria will be maintained at a relatively stable state; they participate in physiological functions of the body in various ways, supplement digestive enzymes by secreting enzyme-containing metabolites, promote the digestion, absorption, and metabolism of substances in the body, or accelerate the development of hyper-productive endo-epithelial cells and the regeneration of vascular tissue, stabilize the immune system of the body, and prevent the reproduction and differentiation of pathogenic bacteria [7–9]. Intestinal flora imbalance will lead to a series of clinical symptoms. the disturbance of the dynamic balance of intestinal flora causes the proliferation of pathogenic bacteria and stimulates the intestinal mucosal barrier, which damages the immune system of the body and leads to the decline of immune function. Moreover, the release of a large number of pathogenic bacteria increases the endotoxin in the intestine of the body and damages the intestinal mucosal barrier, leading to intestinal dysfunction [10,11].

Studies have pointed out that intestinal flora dysregulation is one of the development mechanisms of constipation, and constipation may aggravate intestinal flora dysregulation [12]. Intestinal flora imbalance promotes constipation, which is mainly manifested by the increase in pathogenic bacteria in the body and the decrease in dominant flora. Meanwhile, long-term accumulation of intestinal feces in patients with constipation may lead to the reproduction of pathogenic bacteria in the intestine, and then destroy the intestinal mucosal barrier and aggravate intestinal flora imbalance [13]. The neurotransmitter 5-HT is an important cholinergic transmitter secreted and synthesized by the chromaffin cells of the intestinal mucosa. It can improve intestinal permeability, increase intestinal osmotic pressure, increase water secretion, and promote intestinal peristalsis to play an important role in regulating gastrointestinal motility and gastrointestinal secretion function [14]. Studies have shown that the 5-HT transmission pathway is the key factor in the pathogenesis of chronic constipation [15]. SERT is a transmembrane transporter that exists in all intestinal epithelial cells and has a strong affinity for 5-HT. It can ingest and inactivate the excessively accumulated and physiologically effective 5-HT substances in mucosa or neuronal synapses, and participate in mediating gastrointestinal motility. Therefore, the strength of intestinal motility may be related to the abnormal expression of intestinal SERT protein [16]. However, it is not known whether the disorder of SERT is specifically related to the change in the composition of the intestinal microecological environment. At present, the clinical awareness of constipation is limited, and there is still no sufficient evidence on the specific ways and approaches of intestinal flora dysregulation to promote constipation. Therefore, the molecular regulation mechanism of intestinal flora in the pathogenesis of constipation was investigated in this study, as well as a new idea of treating constipation by regulating intestinal flora.

## 2. Materials and methods

### 2.1 Animal model used for the study

In this study, 60 specific pathogen-free (SPF) mice from Xuzhou Municipal Hospital Affiliated to Xuzhou Medical University were selected as the research objects, with half males and half females. Besides, their body weight was 18–23 g, and the mice were reared adaptively under 25 ± 1℃ and 50 ± 5% of humidity. The feeding was strictly following SOP operating procedures, and the mice were free to eat and drink water. Vitals such as body weight, diet, and activity status of mice were monitored every week. In addition, this study was approved by the Ethics Committee of Xuzhou Municipal Hospital Affiliated to Xuzhou Medical University.

### 2.2 Collection and preparation of fecal bacteria liquid

According to the Rome III standard [17]: the feces of patients with chronic constipation are collected. The screening of fecal flora donors requires detailed medical certification, including disease history, related examinations, and laboratory tests including stool and serology. Six patients with chronic constipation were selected, and all the patients had informed consent. Among them, male/female was 3/3, aged 26–49, bowel movements were 0 to 2 times/week, and Bristol score was 1 to 2. Fecal bacteria liquid was prepared with reference to previous literature [18]: an appropriate amount of feces were taken and placed in a blender, PBS solution was added at a ratio of feces/PBS of 1:10 g/ml for preliminary mixing, the fecal slurry was filtered through stainless steel filters of different diameters and large particles in it were removed. The filtered sample was centrifuged at 6000 rpm for 15 minutes to homogenize and separate, and the fecal bacteria liquid after centrifugation was purified. Then, an appropriate amount of glycerin was added to a concentration of 10%, and stored in a refrigerator at −80°C for later use. The effective storage time was 1–8 weeks.

### 2.3 Establishment of the intestinal flora consumption model of mice

A mouse model of constipation was constructed by referring to previous literature [9]: 20 SPF mice were divided randomly into 2 groups. Mice from both groups were given distilled water intragastrically for 7 days, and the weight of each mouse was measured every morning. After the last gavage on the 7^th^ day and mice from both groups fasted for 24 hours, the mice from group A were gavaged with constipation human fecal bacteria solution, and the mice from group B were gavaged with distilled water of the same amount. The frequency of defecation and fecal dry weight and water content of mice were monitored at the first hour. After the monitoring, cervical spine removal was performed and all mice were sacrificed. The mice were dissected layer by layer, the small intestine was cut open for intestinal separation, and the colonic tissue was retained. The phosphate buffer saline (PBS) solution was used to wash the inner side of the intestinal wall of mice, and the colon tissue was cut lengthways. Half of the colon tissue was stored with liquid nitrogen; the remaining colonic tissue was rolled up and fixed on the transverse axis and then placed in 10% formalin for labeling.

### 2.4 Determination of defecation parameters in mice

20 SPF mice were grouped randomly into two groups, and the intervention method was the same as the establishment of the model. Mice from group A were given constipation human fecal bacteria liquid by gavage, while group B was given the same amount of distilled water by gavage. After 1 hour, both groups of mice were intragastrically gavaged with Indian ink (0.6 mL/mouse). After intragastric administration, mice were given a normal diet, and their stool color, grain number, weight, and intestinal motility were recorded and observed. The gastrointestinal transmission time was the time from the end of the intragastric administration of ink to the first black stool.

### 2.5 Small intestinal propulsion rate test

Referring to the test method for the improvement of the inhibition of intestinal peristalsis caused by constipation in the ‘Technical Specification for Health Food Inspection and Evaluation’ (2003 Edition) of the State Food and Drug Administration of China, 20 SPF mice were selected and split randomly into 2 groups, and the intervention method was the same as when the model was constructed. Mice in group A were gavaged with constipation human fecal bacteria solution, and mice in group B were gavaged with distilled water of the same amount. After 1 hour, both groups of mice were intragastrically gavaged with Indian ink (0.6 mL/mouse). 40 minutes later, all mice were sacrificed by neck removal. After laparotomy, the small intestine from the pylorus to the ileocecal part of mice was cut and the intestinal tube was gently pulled to a straight line. The total length of the small intestine and the length of ink pushing in the intestinal tube of mice were measured to calculate the ink propulsion rate of mice. The equation for calculating the propulsion rate of small intestine ink is as follows.

(1) $$Iipr=\frac{l}{l_{0}}\times 100\%$$

In the above equation, Iipr represents the ink advancing rate of the small intestine, l represents the ink advancing length, and $l_{0}$ represents the total length of the small intestine.

### 2.6 In vitro experiments on Caco-2 cells

The Dulbecco’s modified eagle medium (DMEM) cell culture medium was prepared with non-essential amino acids and antibiotics from fetal bovine serum (FBS). Caco-2 cells were cultured at temperature 36℃, with 85% humidity and 5% CO_2_; the DMEM culture medium was changed every 3 days. When the cells grew to 80 ± 5% fusion, digestion was carried out based on trypsin for 10 minutes. After digestion, the cells were inoculated and adhered to the 6-well plate, the number of cells in each well was 8 × 10^4^, and the cells were cultured at 25 ± 2℃. When the cells grew to about 80%, 0.5% FBS was applied to starve the cells for 10 hours. Then, the cells in group A were stimulated with fecal bacteria solution for 4 hours, and the cells in group B were stimulated with distilled water for 4 hours. After the full stimulation, the medium was discarded, the cells were washed twice with PBS, and Trizol reagent was added to lyse the cells. After the lysis, the cells were stored at the low temperature for later use.

### 2.7 Real time-polymerase chain reaction method

#### 2.7.1 RNA extraction

Referring to previous literature for Real time-PCR experiment [19]: a small amount of mouse colon tissue was added into the ribonucleic acid (RNA)-free Eppendorf (EP) tube, and then, 1 mL of Trizol lysate was added for lysis. The cell suspension was obtaining by repeated mixing with an automated pipette. The cell suspension was allowed to stand at room temperature for 5 minutes, 180 μL of chloroform was added, and then, the mixture was shaken thoroughly and mixed. Afterward, it was placed on ice for preservation. After ice treatment, it was quickly transferred to a centrifuge tube for centrifugation. At the end of centrifugation, the upper aqueous phase was discarded and the isopropanol of the same volume was added into the remaining solution. Next, it was shaken fully and stood for 15 minutes. The white precipitates in EP tubes were purified with anhydrous ethanol and centrifuged. The supernatant was discarded, and the diethylpyrocarbonate (DEPC) solvent with a volume ratio of 1:1 was added. The concentration and optical density of the mixture were determined and the RNA content was calculated.

The method of cell RNA extraction was similar to that of tissue RNA extraction. A small amount of mouse colon tissue was added into the EP tube without a nucleic acid nucleus, and then, 1 mL of Trizol lysate was added for lysis. The subsequent operation was the same as that of tissue RNA extraction.

#### 2.7.2 Western blot method

I: The cleaned glass plate was installed tightly and clamped into the glue holder, about 2/3 of the 10% separation glue of the glass plate was poured, distilled water was added to cover the remaining 1/3, and stood at room temperature until the glue was solidified. Then the distilled water was discarded, the concentrated glue was poured onto the edge of the glass plate, a comb with enough sample holes was inserted, and solidified vertically at room temperature.

II: The glass plate was clamped into the electrophoresis tank containing the electrophoresis buffer. The electrophoresis buffer was under the upper edge of the concentrated gel. The comb was poured vertically. A sampler was adopted to suck the electrophoresis buffer and rinse the sample hole with gel accumulation; then, the broken glue was removed, 5 μl of protein marker was added to the leftmost sample well of the gel, according to the measured protein concentration of each sample, an appropriate volume was sequentially added to the sample hole to keep the protein content consistent. Start constant voltage electrophoresis, electrophoresis was conducted at a constant voltage of 80 V until the protein prestained marker was separated or until the sample reached the separation gel, after 15 minutes, the voltage was increased to 150 V and constant voltage electrophoresis was performed until the sample reached the bottom of the separation gel.

III: The PVDF membrane was taken with the same area as the gel and placed in methanol for pre-activation for 5 minutes, and soaked in the transfer liquid together with two pieces of sponge pad and two pieces of filter paper for later use. One layer of sponge pad and one layer of filter paper were spread on the cathode surface of the film transfer tank, the separation glue containing the target strip and internal reference strip from the glass plate was taken out, they were carefully transferred to the filter paper, and the PVDF membrane was spread to avoid the generation of bubbles. Filter paper and sponge pad were laid out in sequence, the transfer membrane plate was closed tightly and membrane was transferred at 100 V on ice for 100 min.

IV: After the transfer of the film, lift off layer by layer, carefully take the PVDF film, and cut the film strip containing the target strip and the internal reference strip. Incubate the membrane strips with antibodies against 5% skimmed milk powder, and shake them slowly on a shaker at room temperature for about 2 hours.

V: Apply the primary antibody: Discard the blocking solution, wash thoroughly with TBST solution, and then separate the internal reference strip from the target strip along the bottom edge of the 55marker. Put them into a 15 ml centrifuge tube containing 3 μl of SERT antibody (l:1000) and 0.6 μl of internal control β-actin antibody (l:5000), respectively, and incubate them in a shaker at 4°C overnight. Apply the secondary antibody: Wash the membrane thoroughly with TBST solution, wash 3 times 5 min/time at room temperature in a shaker at medium speed, then put each membrane into the correspondingly configured secondary antibody solution (ratio 1:5000), and incubate slowly for 60 minutes. Washing: After the incubation is complete, remove the membrane, and wash it in the TBST solution in the same anti-washing method.

VI: The membrane was laid horizontally in the antibody incubation box, 1 mL of A and B luminescent mixture was dropped evenly onto the membrane strip, and stand for 1–2 minutes. The sample gun could be used to gently blow and suck along the position of the protein to make the protein and the luminescent mixture completely contact, and then the sample was placed in the dark box for imaging.

VII: The film was scanned or photographed, and ImageJ software was used to analyze the protein grayscale of each band on the film.

### 2.8 Enzyme-linked immunosorbent assay

ELISA experiments were performed with reference to previous literature [20]. After weighing, part of the mouse colon tissue was fully ground and transferred to an EP tube with PBS. After the skin was prepared, the radio immunoprecipitation assay (RIPA) cracking homogenate was added to it. After it was fully homogenated, the homogenate was quickly transferred to the centrifuge tube. It was incubated at a controlled temperature of 0℃ for 20 minutes and centrifuged for 3 minutes (8,000 r/min). After centrifugation, part of the supernatant was aspirated with a pipette, and the centrifugal liquid was diluted according to the concentration gradient of 180 ng/mL, 120 ng/mL, 70 ng/mL, 35 ng/mL, and 15 ng/mL. After the dilution, a sample was added to each well. 60 μL was sampled to each well, and the blank well was not added with the sample. After samples were added, the polyvinylidene fluoride (PVDF) sealing plate membrane was used for sealing, which was incubated on a 38 shaker at constant temperature for 1 hour. The sealing plate membrane was torn off layer by layer, the supernatant was discarded, and the sealing plate membrane was dried at room temperature. PBS solution was added for washing 3 times, and the chromogenic agent was added at 38℃ for color development. After the color development, the blank well was set to zero; the absorbance of the samples with various concentrations was calculated, as well as the linear regression equation of the standard curve.

### 2.9 Immunofluorescence staining

The remaining colon tissue stored in 10% formalin was taken and washed with anhydrous ethanol 3 times to dehydrate and soaked in new xylene. After soaking and transparency, the colon tissue was transferred to the 55–60 soft wax and soaked in wax for 30 minutes, which was repeated twice. After paraffin impregnation, the model was assisted with paraffin impregnation and cooled at room temperature. The thickness of the tissue sections was adjusted to 3 μm; the sections were placed on glass slides drenched with a little distilled water and heated with an alcohol lamp until the slides were spread and cooled at room temperature for later use.

The successfully prepared paraffin sections were dewaxed with xylene and hydrated with gradient ethanol. The sections were treated with 5% hydrogen peroxide at room temperature for 5 minutes and heated in a microwave oven to repair antigen. After the repair, the sections were cooled to room temperature and washed with PBS solution 3 times; the residual PBS washing solution was dried with filter paper, and then, the cells were sealed with PBS. After 1 hour of sealing, the blocking fluid was poured out, and the fluorescent primary antibody and secondary antibody were added for incubation; the incubation was done at room temperature in the dark for 2 hours. At the end of incubation, PBS was used for washing 3 times, and then, the excess PBS was absorbed and discarded. Finally, the membrane was sealed and placed in the fluorescence microscopic imaging system (KEYENCE) for imaging.

### 2.10 Diversity index analysis

The Alpha diversity index was adopted to analyze the abundance and diversity of the microbial community, including the Chaol index, Whole Tree index, Shannon index, and Simpson index. The greater the value of the four indicators, the higher the diversity of the microbial community. Among the four indicators, the Chaol index and Whole Tree index reflected the abundance of community species in the sample, and were commonly used to estimate the total number of species; Shannon index and Simpson index were used to quantitatively describe the biodiversity of a region or community in ecology. The equation for calculating the Chaol index was as follows:

(2) $$S_{chao}=S_{obs}+\frac{n\left(n-1\right)}{2\left(n{\;}^{\prime }+1\right)}$$

In the above equation, $S_{chao}$ represents the estimated number of OUTs (Operational Taxonomic Unit), $S_{obs}$ represents the number of observed OUTs, n represents the number of OUTs with only one sequence, and $n{\;}^{\prime }$ represents the number of OUTs with only two sequences.

The Whole Tree index represents the sum of the shortest evolutionary branch lengths of all species in a certain location to the total branch length of each node. Shannon is the flora diversity index, and its calculation equation is as follows:

(3) $$H=-\sum \left(P_{i}\right)\left(\ln P_{i}\right)$$

In the above equation, H represents the total number of samples, and $P_{i}$ represents the proportion of individuals belonging to the i-th species in the sample. The more even the distribution of individuals among different species, the greater the value of H.

The Simpson index represents the probability that two randomly sampled individuals belong to different species, and its calculation equation is as follows:

(4) $$D_{simpson}=\frac{\sum_{i=1}^{S_{obs}}n_{i}\left(n_{i}-1\right)}{N\left(N-1\right)}$$

In the above equation, $S_{obs}$ represents the number of OUTs observed, $n_{i}$ represents the number of OUTs containing i sequences, and N represents the number of all sequences.

### 2.11 Microbial sequencing of fecal flora

Referring to previous literature for microbial sequencing [21], fecal deoxyribonucleic acid (DNA) was extracted by referring to the extraction method of QIAAMP Fast DNA Stool Mini Kit. The concentration of fecal DNA was determined by gel electrophoresis experiment, and the total DNA mass was measured by ultraviolet spectrophotometer. Then the diluted genomic DNA was taken as a template to perform microbiome sequencing. The specific operations were PCR amplification and product purification for the V3-V4 region of the sample DNA, tapping and recovering the target bands to obtain purified specimens. A microplate reader was used to measure each sample, and the IlluminaTruSeq standard DNA library was used for the construction of the computer library. Finally, the rDNA gene sequencing was performed using an MiSeq. At the end of sequencing, the original data was sorted and filtered, and the quality was evaluated. The obtained sequences were merged with 97% similarity and operability classification units were divided and analyzed for diversity using QIIME software, so as to obtain the results of changes in fecal flora of mice in groups A and B.

### 2.12 Statistical analysis

SPSS22.0 software was used for statistical analysis of the data in this study. The count data were represented as % and tested by $\chi^{2}$. The t-test was used between two independent samples, and *P* < 0.05 indicated that the difference was statistically substantial.

## 3. Results

The imbalance of intestinal flora promotes constipation, which is mainly manifested by the increase of intestinal pathogenic bacteria and the decrease in dominant flora. SERT has a strong affinity for 5-HT, so the strength of intestinal motility may be related to the abnormal expression of SERT protein, and the dysregulation of SERT may be related to changes in the composition of the intestinal microecological environment. Therefore, this research constructs a mouse constipation animal model and conducts in vitro cell experiments to study the molecular regulation mechanism of intestinal flora in the pathogenesis of constipation.

### 3.1 Influence of intestinal flora imbalance on defecation function of mice

**Figure 1.**
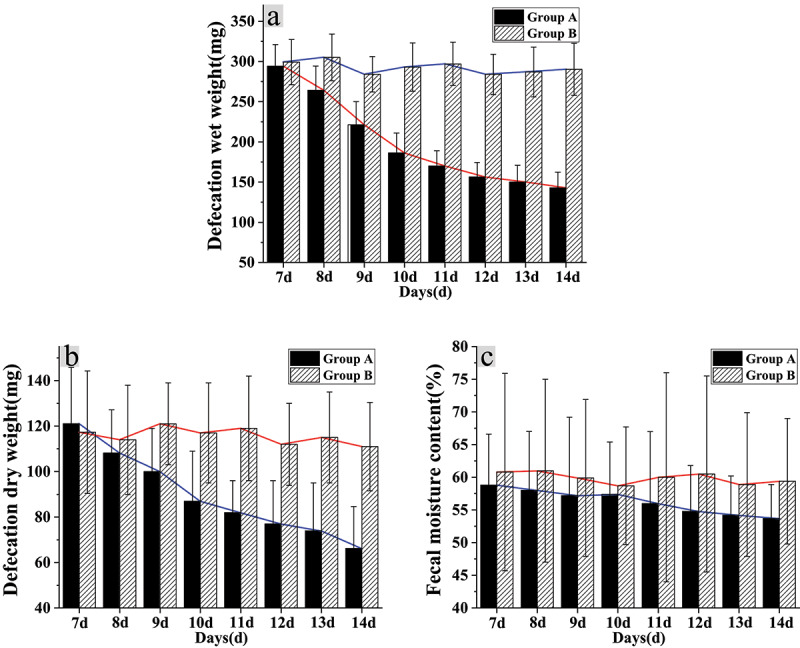
Influence of intestinal flora imbalance on defecation function of mice

After intragastric administration of distilled water on the 7^th^ day, the wet defecation weight of mice from group A was 294.17 ± 26.9 mg, the dry defecation weight was 121.09 ± 24.81 mg, and the fecal water content was 58.8 ± 7.8%. In group B, the wet defecation weight was 299.21 ± 28.12 mg, the dry defecation weight was 117.32 ± 32.38 mg, and the fecal water content was 60.8 ± 15.1%. Therefore, there were no marked differences in the wet defecation weight, the dry defecation weight, and the fecal water content between the two groups on the 7^th^ day of the experiment (*P* > 0.05). After intragastric administration of constipated people’s feces and distilled water, the defecation parameters of mice in the two groups were detected on the 14^th^ day of the experiment. It was found that the wet defecation weight of mice in group A was 142.89 ± 19.45 mg, the dry defecation weight was 66.21 ± 18.44 mg, and the water content of feces was 53.7 ± 5.2%. The wet defecation weight, dry defecation weight, and fecal water content of mice from group A were lower sharply than those of group B on the 14^th^ day of the experiment, and the differences were statistically obvious (*P* < 0.05) (Figure 1).

(Note: A: The wet weight of feces; B: The dry weight of feces; C: The water content)

### 3.2 Ink propulsion rate test of the small intestine

**Figure 2.**
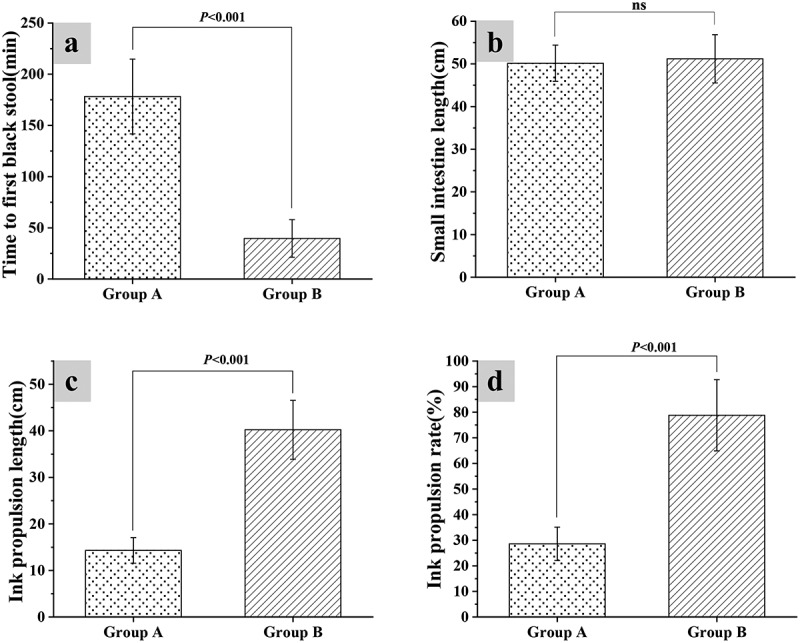
Ink propulsion test results of the small intestine of mice

Mice from group A and group B were given fecal fluid and distilled water for constipation, respectively. By analyzing the effect of constipation on the propulsion rate of intestinal ink in mice, it was found that the first time of black stool in group A was 178.22 ± 36.49 minutes, and the first time of black stool of group B was 39.61 ± 18.35 minutes, so the time of the first black stool in group A was hugely different from that of group B (*P* < 0.05). The total length of the small intestine, the ink propulsion length, and the small intestine propulsion rate were recorded after all mice were humanely euthanized by decapitation under anesthesia. It revealed that the total length of the small intestine from group A was 50.15 ± 4.23 cm, which was not significantly different from the length of group B (*P* < 0.05); the small intestinal propulsion rate of group A was 28.6 ± 6.5% and the ink propulsion length was 14.32 ± 2.76 cm, while the small intestinal propulsion rate and ink propulsion length of group B were 78.8 ± 13.9% and 40.22 ± 6.31 cm in turn. The difference between the ink propulsion length and small intestinal propulsion rate of group A and group B was statistically significant (*P* < 0.05) (Figure 2).

(Note: A: The time of the first defecation; B: The total length of the small intestine; C: The ink propulsion length; D: The ink propulsion rate.)

### 3.3 Changes in intestinal flora diversity after constipation

**Figure 3.**
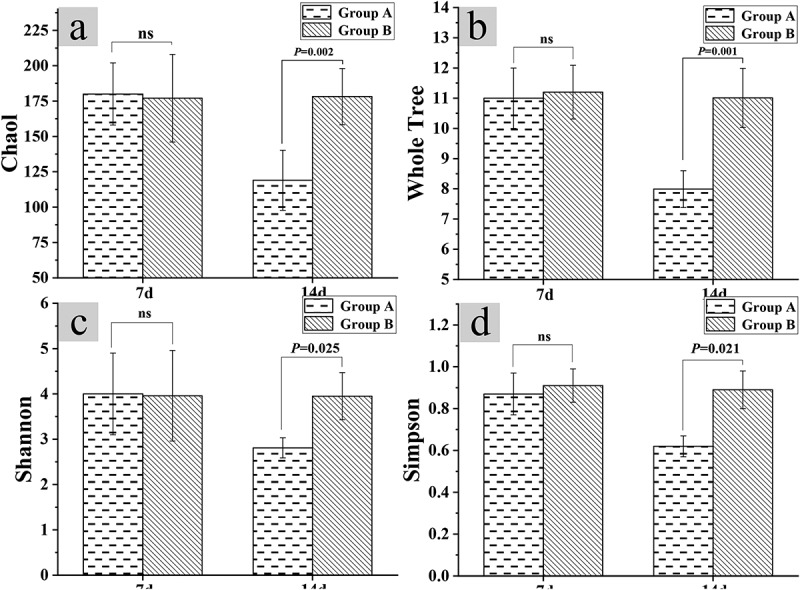
Relationship between constipation and intestinal flora diversity

After the test for 14 days, the four indicators of Chaol index, Whole Tree index, Shannon index, and Simpson index were used to detect the diversity of intestinal flora of mice in groups A and B. The test results of the diversity of the intestinal flora of the mice in groups A and B were shown in Figure 3. The results disclosed that the Chaol index, Whole Tree index, Shannon index, and Simpson index of mice in group A were 118.98 ± 21.23, 7.99 ± 0.61, 2.81 ± 0.22, and 0.62 ± 0.05, respectively. What’s more, the Chaol index, Whole Tree index, Shannon index, and Simpson index of group B were 178.15 ± 19.89, 11.01 ± 0.98, 3.95 ± 0.52, and 0.89 ± 0.09 in sequence. Thus, the above four indexes of group A decreased steeply in contrast to those of group B (*P* < 0.05).

### 3.4 Relationship of intestinal flora dysregulation with intracellular mRNA and protein expression

**Figure 4.**
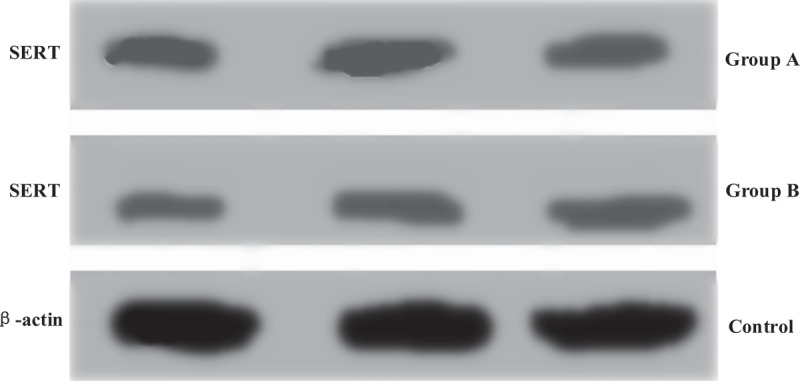
Grayscale analysis results of mouse protein expression level

**Figure 5.**
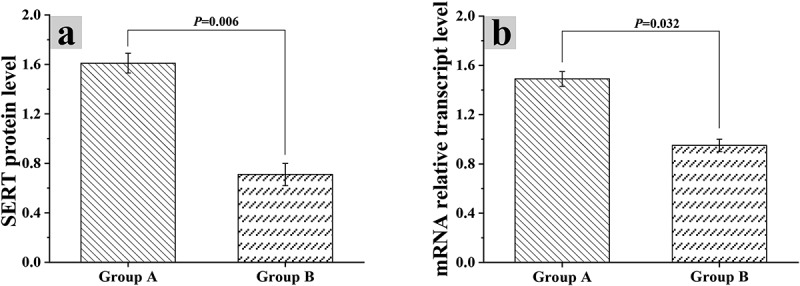
Influences of intestinal flora on SERT mRNA and protein expression levels in Caco-2 cells

The protein expression levels of mice in group A and group B were analyzed by gray-scale analysis, the mRNA levels in Caco-2 cells were analyzed by in vitro experiment with Caco-2 cells, and the results were shown in Figure 4 and Figure 5. The SERT mRNA level in Caco-2 cells of group A was 1.61 ± 0.08, and its relative expression was 1.49 ± 0.06. The SERT mRNA level in Caco-2 cells of group B was 0.71 ± 0.09, and the relative expression level of SERT mRNA was 0.95 ± 0.05. Based on the above, there were statistically great differences in the two indexes between group A and group B (*P* < 0.05)

### 3.5 Relationship between ELISA and 5-HT in mice colon

**Figure 6.**
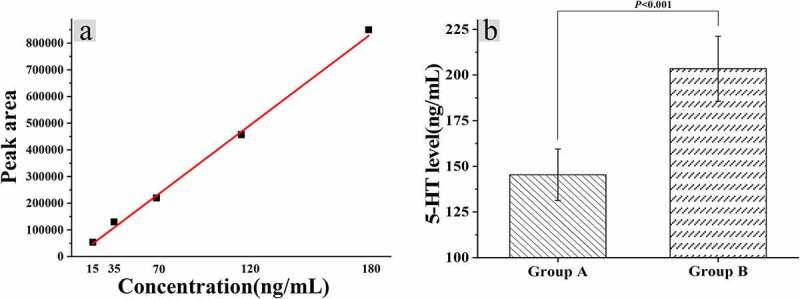
Relationship between dysregulation of intestinal flora and 5-HT levels

The levels of 5-hydroxytryptamine (5-HT) in colonic tissues of mice were calculated after the fecal fluid of constipated people was given gavage with distilled water, and the standard curve was drawn according to the peak area corresponding to the centrifuge with different dilution concentrations, as shown in Figure 6a. Furthermore, the standard curve regression equation was calculated as follows: y = 2.36105e-004x+4.13906, r = 0.9989. The 5-HT level in the colon tissue of mice in group A was calculated to be 145.36 ± 14.12 ng/mL, and the level of group B was 203.44 ± 17.81 ng/mL, and the difference between the two groups was statistically huge (*P* < 0.05).

### 3.6 Immunofluorescence staining results

**Figure 7.**
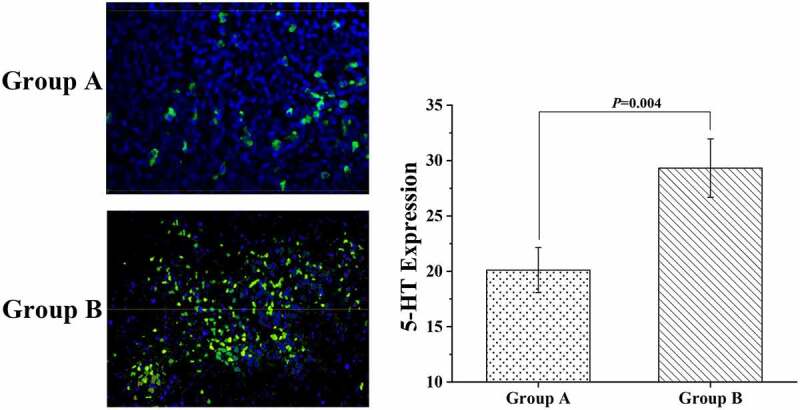
The results of the immunofluorescence staining experiment

After fluorescence staining, the colonic tissue samples of mice were placed under a microscope to observe the distribution of 5-HT in the samples. The results were displayed in Figure 7, suggesting that the differences in 5-HT expression in this study were mainly found in the epithelial cells of the colon surface of mice, and the expression of 5-HT in the epithelial cells of the crypt site was relatively small. The expression level of 5-HT was 20.11 ± 2.03 in group A and 29.32 ± 2.64 in group B, indicating a significant difference between the two groups (*P* < 0.05).

(As shown in the figure, in immunofluorescence double soft color detection of 5-HT expression in mouse colon tissue, 5-HT appears green under blue fluorescence)

### 3.7 Microbial sequencing results of fecal flora

**Figure 8.**
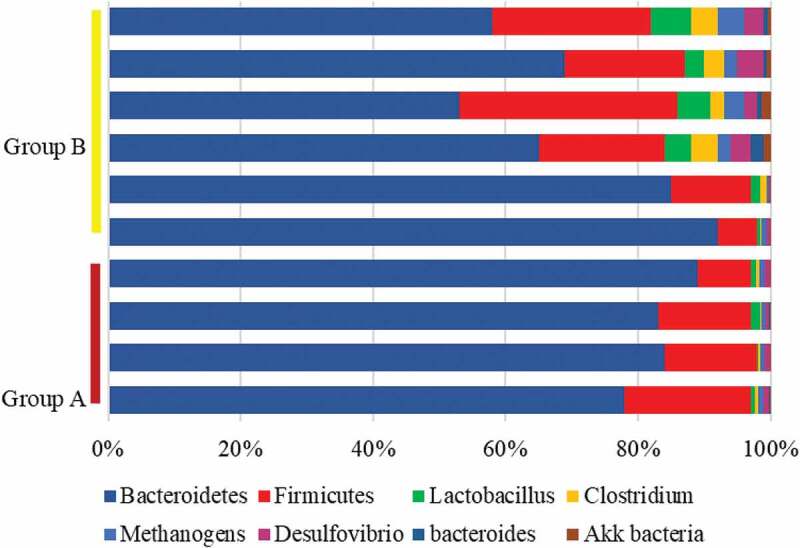
Changes in intestinal flora levels of mice from the two groups

The intestinal flora of mice from both groups was sequenced using the sequencing platform. The results revealed that the number of Lactobacillus, Clostridium, Methanogen, and Desulfuribrio in the intestinal flora of mice from group A reduced hugely compared with group B, while the number of Akk and Bacteroidetes rose substantially in contrast to those of group B, with statistical significance (*P* < 0.05). At the phylum level, the proportion of Firmicutes in the intestinal tract of mice from group A was lower sharply than the proportion of group B, and the proportion of Bacteroidetes was higher hugely than that of group B (*P* < 0.05) (Figure 8).

## 4. Discussion

In the human intestine, various microorganisms are form the intestinal flora. The intestinal bacterial biofilm can not only cause the malignant transformation of intestinal epithelial cells by triggering the host’s persistent inflammatory response, but also exert carcinogenicity by inhibiting the growth of intestinal microorganisms [22]. Studies have found that there is a general imbalance of intestinal flora in the intestines of people with constipation, that is, disorders of intestinal flora may lead to constipation. The results of this study found that the imbalance of intestinal flora would cause abnormal bowel function in mice, cause abnormal intestinal motility in mice, and reduce the diversity of microbial flora in the intestinal tract of mice. Gray-scale analysis results showed that the SERT mRNA level and relative expression in Caco-2 cells of mice in the constipation group were significantly higher than those in the distilled water group, the 5-HT level was lower than the distilled water gavage group, and the 5-HT expression on the surface of the biofilm-positive colon tissue was higher than that of the biofilm-negative colon tissue. 5-HT is an important cholinergic transmitter, mainly secreted by enterochromaffin cells, which are very sensitive to external stimuli. When the intestine is stimulated, enterochromaffin cells will release 5-HT, and then gather with the subtypes of 5-HT receptors to play a physiological role [23]. SERT is a transmembrane transport protein that exists on the enterochromaffin cell membrane and the presynaptic membrane. It has a high affinity for 5-HT and a powerful 5-HT transport function. It can quickly re-uptake the 5HT accumulated in the effect site into chromaffin cells or nerve endings, so as to terminate the physiological effects of 5-HT in time. SERT gene defects can lead to significant changes in the diversity and richness of pathogenic bacteria or probiotics associated with colitis, and at the same time promote inflammation and increase the susceptibility to intestinal inflammation [24]. This study found that the expression of SERT in mouse colon tissue would increase with the imbalance of intestinal flora, and the 5-HT level in colon tissue was negatively correlated with gastrointestinal emptying time. In other words, SERT on the intestinal bacterial biofilm down-regulated the expression of 5-HT in the epithelial cells of the mouse colon tissue, thereby changing the intestinal motility.

More and more studies have shown that constipation is not only related to diet structure and intestinal dynamics, but also often accompanied by imbalance of intestinal flora. According to physiological functions, the intestinal flora can be divided into probiotics, pathogenic bacteria, and pathogenic bacteria. With the development of 16s rDNA technology, DNA sequencing methods can accurately determine and analyze the types and structural composition of intestinal flora [25]. The results of this study showed that the numbers of Lactobacillus, Clostridium, methanogens, and Desulfovibrio in the intestinal flora of constipated mice were significantly lower than those in the healthy control group (P < 0.05). but Bacteroides and Akk were significantly increased (P < 0.05). Lactobacillus is a typical obligate anaerobic bacteria that can ferment and degrade in the intestine, lower the pH of the intestine, and produce metabolites including short-chain fatty acids. Short-chain fatty acids have the effect of lowering intestinal pH, promoting colonic peristalsis, and reducing the transit time of feces in the colon. The low pH environment also uses positive feedback adjustment to promote the fixed value of obligate anaerobes in the intestine, inhibit the fixed value and reproduction of conditional pathogens and pathogenic bacteria, improve the intestinal flora disorder of patients with constipation, and maintain the structure of normal intestinal flora [26]. Clostridium belongs to conditional pathogenic bacteria. The accumulation of feces triggers the proliferation of pathogenic bacteria, releases endomycin to destroy the intestinal mucosal barrier function, and then secondary related inflammatory factors aggravate the intestinal flora imbalance [27]. Different from previous studies, this study observed that the Clostridium in the control group was higher than that in the constipation group, indicating that the upregulation of Clostridium was related to the increase in intestinal motility. Therefore, it is speculated that Clostridium may promote intestinal motility through 5-HT, so the level of Clostridium in the intestine of constipated mice is low. The abundance of methanogens is positively correlated with the severity of constipation symptoms. The abnormal increase of methanogens in the intestine may affect the metabolites in the intestine. Therefore, it is speculated that the methane produced by the intestinal flora may belong to a biologically active molecule that affects the intestinal muscular system through a certain pathophysiological mechanism to interfere with the occurrence of chronic constipation. Studies have shown that methanogenesis will increase the absorption of short-chain fatty acids by the body [28]. In this study, it was observed that the methanogens in the intestines of healthy mice were higher than those in the constipation group, which indicated that the low levels of methanogens in the intestines of constipated mice reduced the absorption of short-chain fatty acids, thereby inhibiting intestinal peristalsis. In the constipation group, the desulfovibrio bacteria in the intestines of the mice were lower than those in the control group, which indicated that desulfovibrio bacteria were the dominant flora in the intestinal tract of mice, and it was colonized in the intestine to intervene in the occurrence of constipation. Finally, by analyzing the mean level and phylum level of the fecal flora of the two groups of mice, it was found that the Akk and Bacteroides in the intestinal tract of constipated mice were much lower than those of healthy mice (P < 0.05), it was suggested that the regulation of short-chain fatty acids by Bacteroides was a positive feedback effect. Akk bacteria is a Gram-negative anaerobic bacteria that can produce a variety of mucin degradation-related proteins. It has the functions of maintaining the intestinal microecological balance, maintaining the intestinal mucosal barrier function, and inhibiting inflammation. At the same time, it can promote the secretion of antimicrobial peptides, increase the thickness of intestinal mucus, and reduce the expression of pro-inflammatory factors, thus playing the most important role in improving the intestinal function of patients with constipation.

## 5. Challenges, needs, and future outlooks

Chronic constipation is a ubiquitous functional intestinal disease. The symptoms are repeated and persistent and difficult to treat, which seriously affects the quality of life of patients. The microbial flora distributed in different stages of the human intestine plays an important role in the occurrence of human health and diseases. The results of this study show that the expression of SERT and 5-HT can change intestinal motility, and the imbalance of intestinal flora may destroy the intestinal barrier function. This experiment explored the possible effects and pathways of intestinal flora imbalance in the occurrence of chronic constipation from different experimental angles and using a variety of experimental methods. However, this study only verified the correlation between changes in intestinal microflora and constipation in animal constipation model experiments, and its regulatory effect on human constipation symptoms still needs further clinical trials. In addition, due to the differences in the spatial distribution of intestinal bacterial biofilms, the biochemical characteristics of the biofilms and the related mechanisms of constipation still need to be further explored. At the same time, due to limited conditions, the relationship between the intestinal flora and the clinical symptoms and symptoms of constipation has not been able to be correlated and remains to be further studied.

At present, clinical research on the relationship between constipation and intestinal flora is still in its infancy. Past studies at home and abroad have shown that patients with constipation generally have an imbalance of intestinal flora, and the imbalance of flora can also aggravate constipation. In the future, Fecal microbiota transplantation (FMT) is an important research direction. FMT is the healthy flora in the feces of transplanted donors, and most of them use the supernatant of fermented feces, fresh feces and pediatric feces. After self-study reported that the infection of Fusobacterium was closely related to the use of antibacterial drugs, FMT was taken seriously. However, the current related clinical studies of this method mostly focus on recurrent Clostridium difficile infection, and there is still a lack of a large amount of clinical data for the treatment of constipation. Therefore, this method can be further discussed in future research.

## 6. Conclusion

In this study, the relationship between intestinal flora dysregulation and constipation in mice mediated by biofilm was investigated by in-vitro cell experiments and an in-vivo constipated mouse model. The results showed that after the occurrence of constipation, the diversity of intestinal microflora decreased, and the probiotics were also decreased. Intestinal microflora dysregulation would lead to the increase of SERT expression level and the decrease in 5-HT in mice with abnormal defecation function and intestinal motility, which would result in the change of intestinal motility and the destruction of intestinal mucosal protective barrier, and finally, constipation was caused. The results of this experiment suggested that the occurrence of constipation could be improved by adjusting the balance of intestinal flora, increasing the diversity of flora, and reducing the genus of opportunistic pathogens.

## Highlights

(1) Intestinal flora promotes chronic constipation by increasing expression of SERT.

(2) Clostridium promotes intestinal motility by up-regulating 5-HT.

(3) The Akk strain can protect the intestinal mucosal barrier and inhibit inflammation.
